# Development of Bio-Composites from Milkweed Fibers Using Air-Laid Spike Process for Automobile Dashboard Applications

**DOI:** 10.3390/ma18030618

**Published:** 2025-01-29

**Authors:** Deborah Lupescu, Patrice Cousin, Mathieu Robert, Said Elkoun

**Affiliations:** 1 Department of Civil Engineering, Université de Sherbrooke, 2500, Boulevard de l’Université, Sherbrooke, QC J1K 2R1, Canada; patrice.cousin@usherbrooke.ca (P.C.); mathieu.robert2@usherbrooke.ca (M.R.); 2 Department of Mechanical Engineering, Université de Sherbrooke, 2500, Boulevard de l’Université, Sherbrooke, QC J1K 2R1, Canada; said.elkoun@usherbrooke.ca

**Keywords:** bio-composites, milkweed fibers, polylactic acid fibers, dashboards, thermal conductivity, sound resistance, hydrophobic material

## Abstract

This study focused on examining the reinforcement of milkweed fibers in polylactic acid (PLA) bio-composites used for dashboards in car interiors. Milkweed fiber is a natural fiber with a hollow structure that provides tremendous thermal insulation and noise resistance properties. Firstly, the milkweed fibers were blended with PLA fibers in a weight ratio of 75:25 using an air-laying process. Then, several layers of nonwoven material were compressed in a hydraulic press to obtain bio-composites. Finally, three bio-composites were obtained with different numbers of layers. The density, microstructure, thermal conductivity, sound transmission loss (STL), mechanical properties, dynamic mechanical analysis (DMA), and contact angles of the bio-composites were evaluated. The microstructure analysis revealed that some milkweed fibers collapsed due to the high-pressure molding process, which does not affect the bio-composite properties. The bio-composite with a higher number of nonwoven layers presented a poor interface between PLA and milkweed fibers, thus making it less homogeneous. This bio-composite showed a decrease of 5% in thermal conductivity values and a 19% increase in STL values. In addition, it exhibited a 160% increase in specific flexural strength and a 335% increase in specific flexural modulus compared to samples with a lower number of nonwoven layers. Therefore, it offers the best mechanical-property-to-density ratio, with values that conform to the specifications required for automotive dashboards.

## 1. Introduction

The automotive manufacturing industry is a major source of greenhouse gas emissions and consumption of non-renewable resources. For this reason, several studies have been conducted on the development of natural-fiber-reinforced thermoplastic matrices in the automotive sector [1,2,3,4]. Indeed, they present numerous advantages such as low cost, low weight, low food carbon emissions, and good mechanical properties. Given their characteristics, composites with natural fibers and thermoplastics used for vehicle interiors, such as door panels, roofs, headliners, front fenders, tailgates, seat backs, package trays, dashboards, and trunk liners, occupy an important place [3,5]. Among these thermoplastic matrices, polypropylene (PP) is one of the most employed due to its numerous properties: low density, low cost, good mechanical properties, good dimensional stability, and flame resistance [2,3,6,7,8]. However, thermoplastic matrices are derived from petroleum resources and require high energy for processing production [9]. They could be replaced by eco-friendly materials made from natural fibers or biopolymers that require less energy than other thermoplastics and come from renewable resources, such as polylactic acid (PLA) [10,11,12].

PLA is a compostable and recyclable polymer produced from the fermentation of polysaccharides extracted from corn, beets, or tapioca [10,12]. PLA exhibits good stiffness and strength, and it demonstrates better tensile properties than polystyrene (PS) and polyethylene terephthalate (PET) [13,14]. For all these reasons, PLA is used as a matrix in bio-composites reinforced with natural fibers such as flax and kapok [10,12]. In fact, bio-composites offer reduced weight, are easy to recycle, and provide advantages in thermal and acoustic insulation performance, along with CO₂ neutrality [15]. The use of bio-composites can reduce the weight by 10% and decrease energy consumption by up to 80% compared to synthetic material composites [12]. However, they present some limitations due to their morphological irregularities, which result in poor mechanical properties and a hydrophilic nature, leading to a weak interface between the matrix and fibers [12]. Indeed, a composite made of a hydrophobic matrix and a hydrophilic fiber results in a heterogeneous composite, leading to lower mechanical properties due to poor affinity [8].

Nevertheless, this can be countered by hydrophobic natural fibers, such as kapok or milkweed fibers. The production of these fibers also presents a low carbon footprint [14]. Thus, to be respectful of the environment, the fibers should come from the same country where the manufacturing of the material takes place. This North American native plant presents a hollow structure with a large lumen and low density, which leads to excellent thermal insulation capabilities and noise-resistance properties [16,17,18,19,20,21,22,23] Unlike other cellulosic fibers, milkweed is hydrophobic due to the waxes and lignin on its surface [19,20,24]

There are few studies that have been conducted on the properties of composites reinforced with milkweed fibers [18,25,26,27]. Reddy et al. studied the mechanical properties of two composites: one made of milkweed and PP and the other composed of kenaf and PP. They found that the milkweed-and-PP composite exhibited better flexural and tensile properties at the same weight ratio and better rigidity than the kenaf-and-PP composite due to the milkweed’s hollow structure [26]. By the same token, Robert et al. found better flexural properties for a milkweed-/PP-carded composite than for a flax/PP composite [25]. They found a 76% increase in the flexural modulus for the same mass ratio compared to the flax/PP composite. However, they found a loss of 52% in the milkweed/PP composite for flexural strength. They claimed that it was due to the lower mechanical properties of raw milkweed fibers compared to those in flax. The sound loss transmission (STL) of nonwoven fabrics made from cotton/kapok and cotton/milkweed was studied by Ganesan et al. They asserted that nonwoven milkweed showed better STL than nonwoven kapok, regardless of the percentages [27]. They claimed this was due to low fiber density, resulting in more surface per unit area.

Whereas composites composed of milkweed fibers have been proven to have better mechanical properties and noise resistance than kapok composites, it is not certain that they are suitable for specific applications such as dashboards. Dashboards must be resistant to wear and tear, thermal stress, and ultraviolet [28]. In addition, they must have a density lower than 1180 kg/m^3^, a tensile strength of at least 25 MPa, and a tensile modulus greater than 2.3 GPa [29]. Moreover, they should be eco-friendly and hazard-free, and in terms of aesthetics, they should have a smooth hand feel and a pleasant visual appearance [28]. Based on these results, bio-composites made from milkweed and PLA fibers have been processed through the air-laying process and then thermocompressed in different Ma.

Several studies have demonstrated the limitations of the carding process with milkweed fibers [30,31,32]. It has been shown by the authors in a previous work that the air-laying process exhibits an overall yield rate of 90%, approximately twice as much as the carding process [33].

The present study investigated whether a 100% hydrophobic bio-composite made of milkweed and PLA fibers can be relevant for use as dashboards for a car interior. For this reason, the Ma, density, microstructure, thermal conductivity, STL, tensile properties, flexural properties, DMA analysis, and contact angle of the bio-composites were evaluated.

## 2. Materials and Methods

### 2.1. Material

Milkweed (MW) fibers were supplied by Monark Cooperative (Notre-Dame-des-Neiges, QC, Canada). Their density was 0.30 g/cm^3^ [16], and the average length, diameter, and thickness measured by a scanning electron microscope (SEM) were 25 ± 3 mm, 22 ± 6 μm, and 1.52 ± 0.21 μm, respectively. PLA was purchased from Ingéo NatureWorks (Blair, NE, USA) Its density was 1.28 g/cm^3^. (Datasheets given from the companies.)

Table 1 shows the properties of the fibers.

The fibers did not undergo any surface treatments since it has been shown that alkaline treatment and the grafting of epoxy silane coupling agents onto the surfaces of milkweed and PLA fibers resulted in fiber collapse and a reduction in mechanical properties [16]. In a previous study, the authors found that the composition of milkweed fibers is 40–45% cellulose, 35–40% hemicellulose, 15% lignin, 3% free sugars, and 3% wax [20].

### 2.2. Bio-Composite Preparation

**Figure 1:**
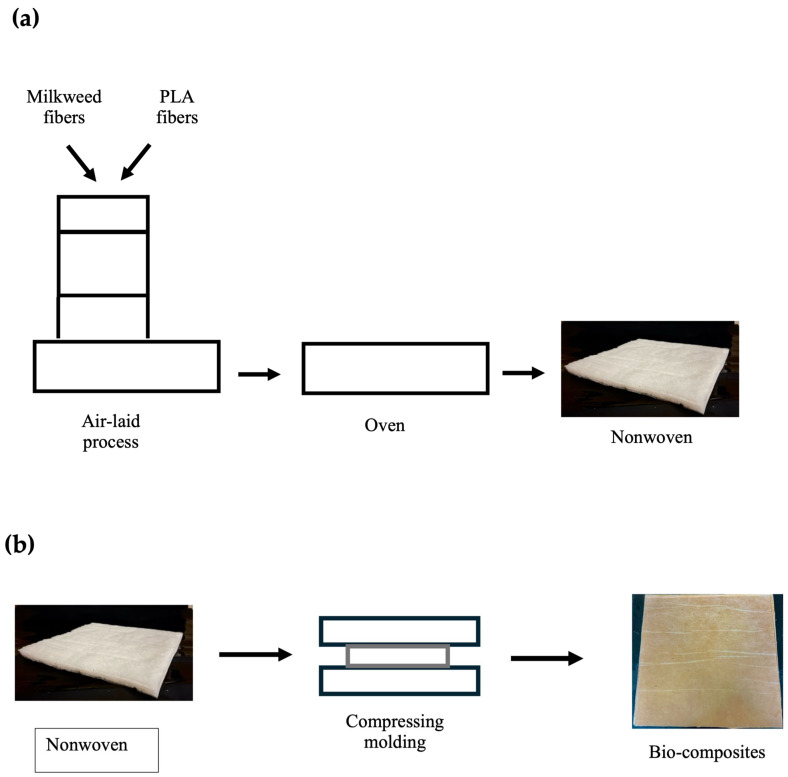
Manufacturing process of (**a**) the nonwovens and (**b**) the bio-composites using air laying.

Bio-composites were produced from nonwovens using the following method. First, the nonwovens were processed using a SPIKE system with milkweed fibers and PLA fibers in a weight ratio of 75/25. Then, the blend was put in an oven to be consolidated at a temperature of 150 °C. A 15 mm thick nonwoven with an Ma of 150 g/m^2^ Ma was obtained. Finally, several layers of nonwoven material were placed in a hydraulic hot press (AutoFour/3012-PL, Carver Inc., Wabash, IN, USA) and pressed at 200 °C for 20 min, allowing the PLA fibers to melt. Three bio-composites were produced. Figure 1a displays a scheme of bio-composite production using the air-laying process. Figure 1b illustrates the production of bio-composites from the nonwoven material through thermocompression.

Table 2 and Table 3 present the bio-composites’ composition and physical parameters, respectively.

### 2.3. Characterization of the Samples

#### 2.3.1. Physical Properties

Density, ρ, was calculated from Equation (1):

(1) $$\rho =\frac{Ma}{TH}$$

#### 2.3.2. Microstructure Analysis of Fibers and Bio-Composites

The morphology of single fibers and bio-composites was studied using a Hitachi S3000-N SEM at 5 kV and 15 kV to determine whether milkweed fibers were damaged by the thermocompression and to assess the dispersion between the fibers.

#### 2.3.3. Thermogravimetric Analysis (TGA)

TGA was performed to investigate the thermal degradation of the bio-composites using a thermogravimetric analyzer (Pyris 4000, Perkin-Elmer, Waltham, MA, USA). All the analyses were conducted on three specimens under oxygen from 50 to 450 °C at 5 °C/min.

#### 2.3.4. Thermal Conductivity

Thermal conductivity was measured using a heat-flow meter (FOX 314, TA Instruments, New Castle, DE, USA) in accordance with ASTM C518-22 at mean temperatures of 29 °C, 5 °C, and −4 °C. The method consists of compressing the bio-composite between two plates at different temperatures with the aim of calculating the heat flow going through.

#### 2.3.5. Sound Transmission Loss (STL)

The STL of the bio-composites was measured using an impedance tube with an internal diameter of 44 mm (Mecanum Instruments, Sherbrooke, QC, Canada). The frequency ranges from 500 to 4500 Hz at a relative humidity of 46% and room temperature according to ASTM E-2611-24.

#### 2.3.6. Tensile Tests

Tensile tests were carried out using a tensile test machine (Z050, Zwick/Roell, Baden-Wuerttemberg, Germany) with a load cell of 30 kN and a crosshead speed set at 1 mm/min in accordance with ASTM D3039. At least 5 measurements were performed. The dimensions of the bio-composite samples were 250 mm × 25 mm.

Specific tensile strength and specific tensile modulus were calculated by dividing tensile strength and modulus by density, respectively.

#### 2.3.7. Flexural Tests

Flexural tests were conducted using a tensile test machine (Z050, Zwick/Roell, Baden-Wuerttemberg, Germany) equipped with a load cell of 30 kN in accordance with ASTM D790-17. At least five samples from each bio-composite were tested. C1 and C2 (50.8 mm × 12.7 mm × 1.40 mm) were tested at a 0.77 mm/min crosshead speed with a 25.4 mm span, whereas C3 (51.1 mm × 12.7 mm × 2.40 mm) was tested at a 1.02 mm/min crosshead speed with a 38.4 mm span.

Specific flexural strength and specific flexural modulus were calculated by dividing flexural strength and modulus by density, respectively.

#### 2.3.8. Dynamic Mechanical Analysis (DMA)

DMA tests were performed in dual-cantilever mode, varying the temperature from −30 °C to 60 °C at a heating rate of 4 °C/min. At least 3 measurements were performed. The dimensions of the bio-composite samples were 60 mm × 10 mm.

#### 2.3.9. Contact Angle

The contact angle was evaluated using a First Ten Angstroms DCA-100 contact angle tensiometer (Newart, NJ, USA). The method followed the evolution of the fiber’s weight during its penetration into a liquid. The contact angle was measured using water, following the Wilhelmy method and in accordance with Equation (2). At least five measurements were performed for each bio-composite.

(2) $$cos\theta =\frac{P}{\gamma_{l}.l}$$

where P is the weight of liquid absorbed by the absorbed fiber, $\gamma_{l}$ is the surface tension of the liquid, and l is the perimeter in contact with the liquid.

## 3. Results and Discussion

### 3.1. Microstructural Analysis

**Figure 2:**
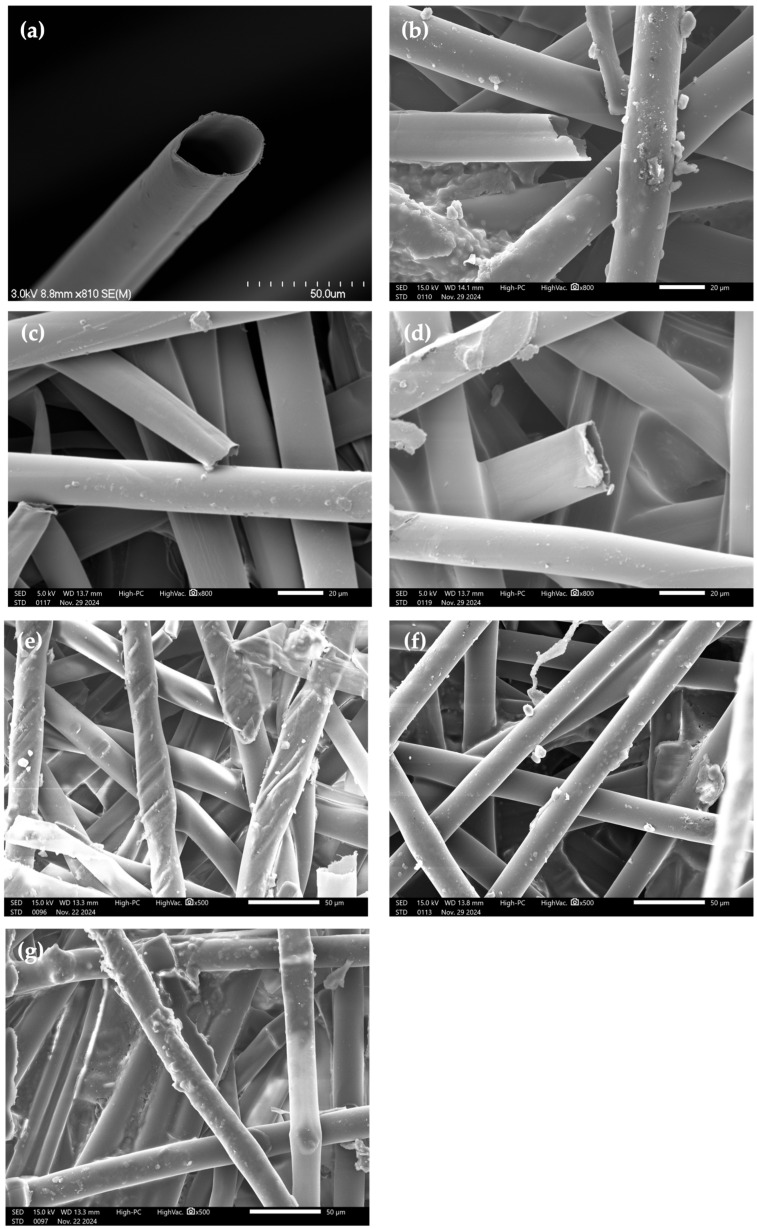
SEM images of (**a**) milkweed fiber and (**b**) milkweed fiber from C1, (**c**) milkweed fiber from C2, and (**d**) milkweed fiber from C3. Dispersion of the fibers in (**e**) C1, (**f**) C2, and (**g**) C3.

Figure 2a illustrates the microstructure of a single milkweed fiber. Milkweed fiber presents a hollow structure with a large lumen and a smooth surface. Figure 2b–d present the microstructure of milkweed fibers taken from samples C1, C2, and C3, respectively. In spite of the thermocompression step, the milkweed fibers kept their hollow structure (Figure 2b–d).

Figure 2e–f compare the dispersion of fibers in samples C1, C2, and C3, respectively. It can be observed that the fibers are better dispersed and interlaced in samples C1 and C2 than in sample C3. This is due to the number of nonwoven layers in each sample, since samples C1 and C2 contain 6 and 7 layers when compared to sample C3, which is composed of 10 layers. In fact, when molded, sample C3’s layers are misaligned due to the applied force.

Therefore, a larger number of overlapped layers leads to a lower fiber dispersion. However, the hollow structure of milkweed fiber is not strongly affected by the high-pressure molding.

### 3.2. Thermogravimetric Analysis (TGA)

**Figure 3:**
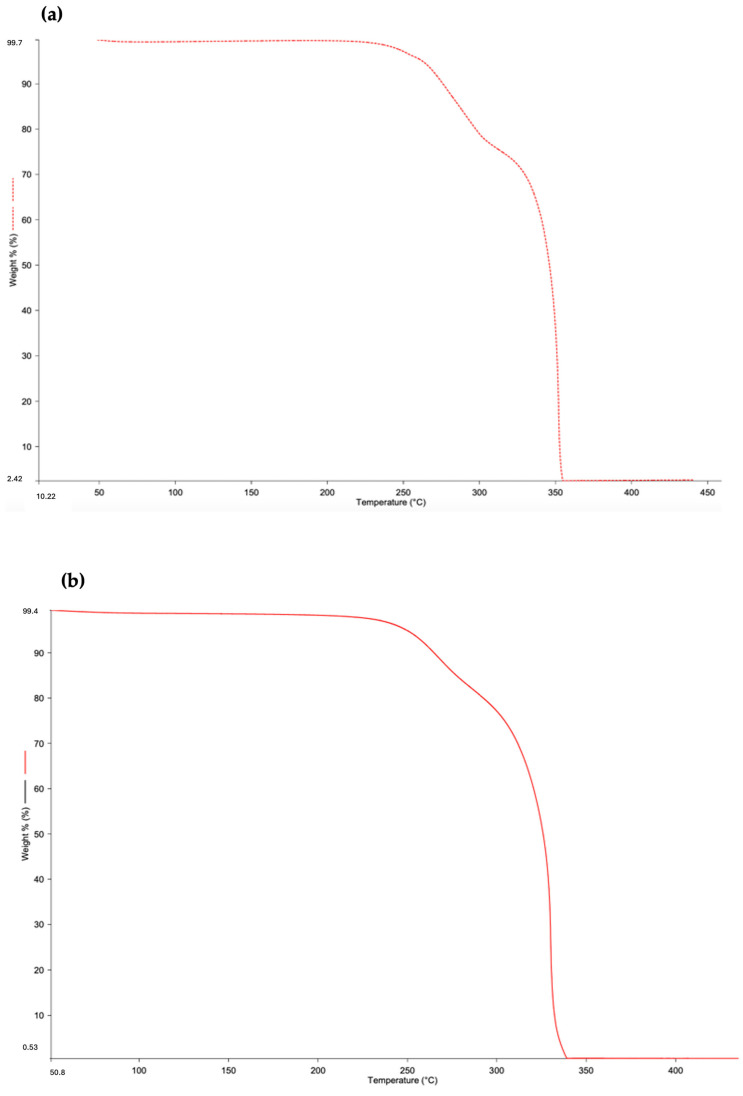

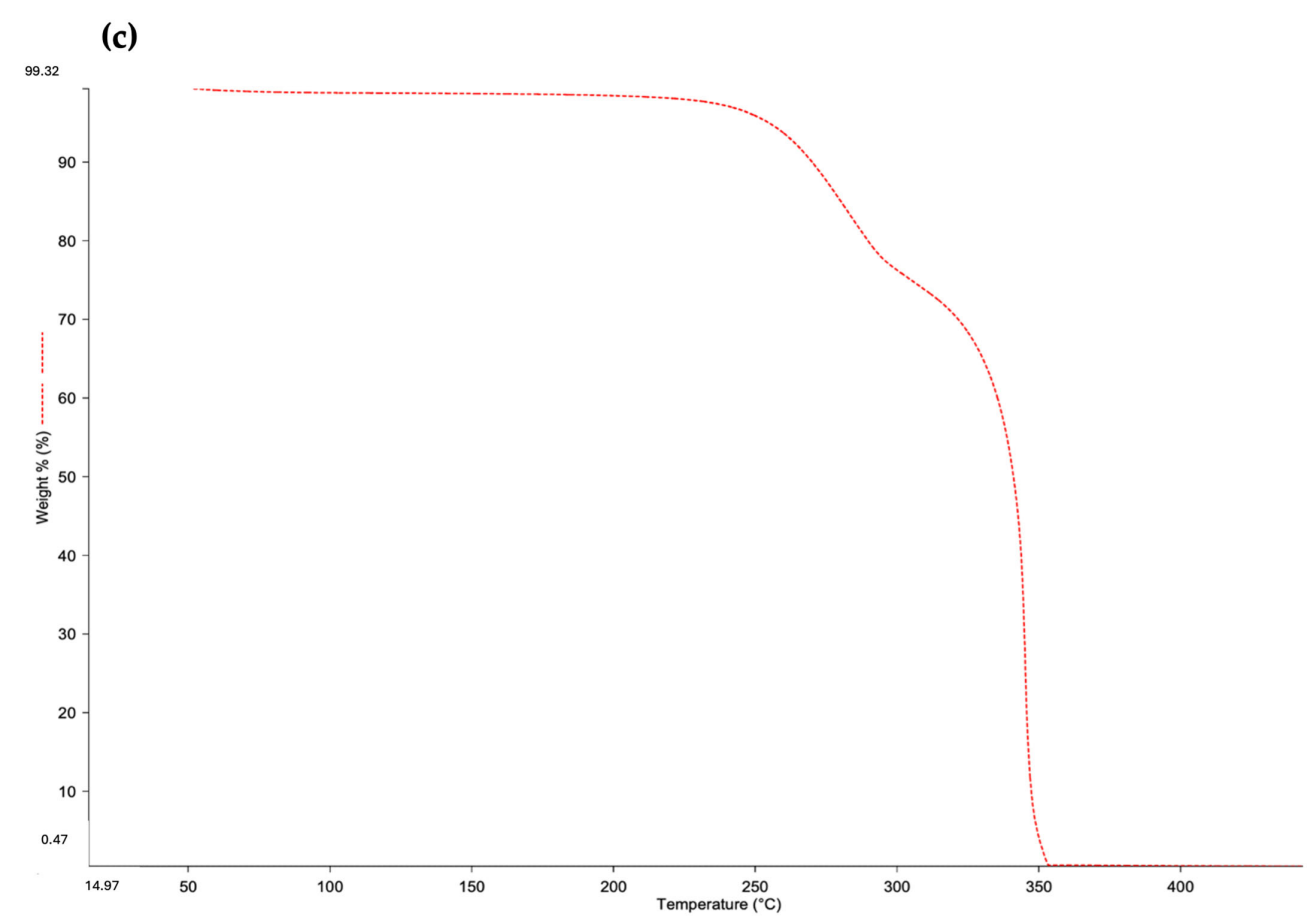
Weight loss curves for (**a**) C1, (**b**) C2, and (**c**) C3.

Figure 3 shows the thermogravimetric analysis for samples C1, C2, and C3.

For all the bio-composites, there was a weight loss of 25%, 20%, and 30% for samples C1, C2, and C3, respectively, at around 240 °C. This weight loss indicates the degradation of hemicellulose and lignin [34,35]. For all the samples, a second weight loss of 75% occurs at around 310 °C, corresponding to the degradation temperature of PLA [36,37].

### 3.3. Thermal Conductivity

Thermal conductivity depends on several parameters, such as density, Ma, and fiber type [38].

**Figure 4:**
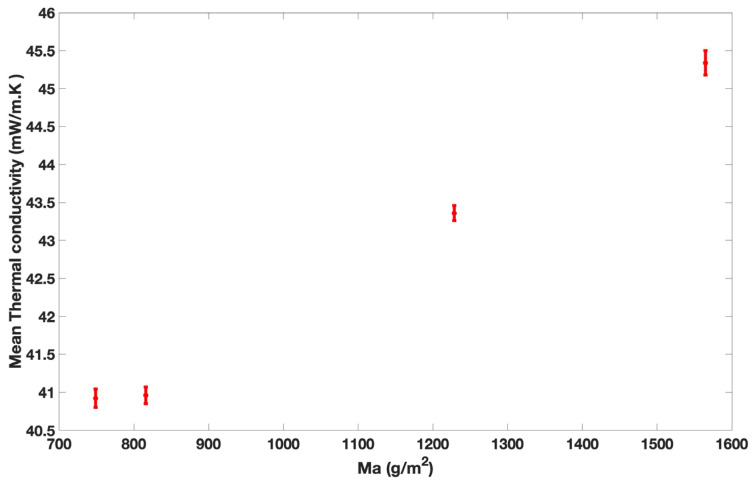
Thermal conductivity values versus Ma.

Figure 4 represents the thermal conductivity values of the bio-composites as a function of Ma.

Thermal conductivity increases with Ma, as mentioned by several studies [39]. This can be explained by the closer contact between fibers, which results in a better heat transfer by conduction [24,38,40]. The thermal conductivity of the bio-composites varies with the mass per unit area (Ma). Samples C1 and C2 present similar Ma values (748.76 g/m^2^ and 815.73 g/m^2^, respectively), which lead to similar thermal conductivity values (40.96 mW/m·K and 40.99 mW/m·K, respectively). Sample C3 exhibits a mass per unit area approximately 64% higher than sample C1 and consequently exhibits a higher thermal conductivity value than C1. Another explanation for this result may be fiber dispersion. Sample C3 is composed of 10 layers, and samples C1 and C2 are composed of 6 and 7 layers; the overlap between layers is more effective in sample C3 as previously mentioned. This finding had already been noted by Song et al., who showed that the thermal conductivity is affected by the dispersion of fibers [41].

Yachmenev et al. studied the thermal conductivity of composites made of bagasse, cotton, and poly(tetramethylene adipate-co-terephthalate) for trunk interiors. They reported thermal conductivity values between 76.2 mW/m·K and 86.4 mW/m·K for a density range varying from 762 to 803 kg/m^3^, which is twice larger than our bio-composites [42]. The thermal conductivity of *Calotropis procera* (another milkweed variety)-fiber-reinforced composites was measured by NagarajaGanes et al. For a fiber content ranging from 10% to 40%, they found thermal conductivity values between 159 mW/m·K and 166 mW/m·K, which are higher than those for samples C1, C2, and C3 [43].

Therefore, even if bio-composites are compressed, they are better insulators than others made of *Calotropis procera*.

**Figure 5:**
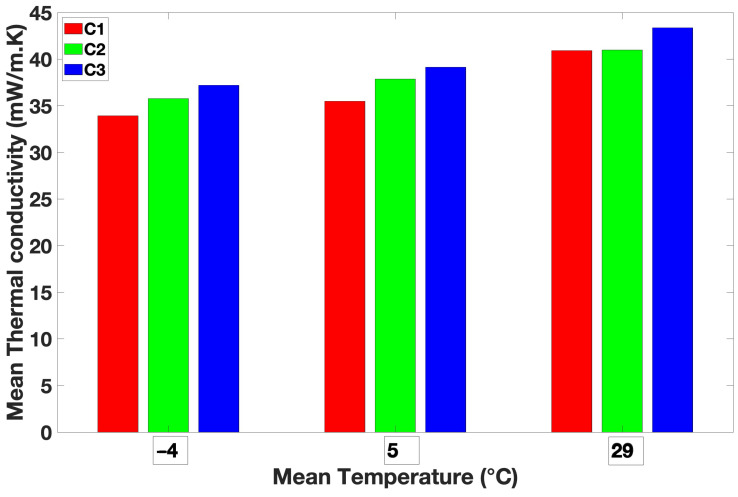
Thermal conductivity values at average temperatures of −4 °C, 5 °C, and 29 °C.

Figure 5 presents the thermal conductivity of bio-composites at various mean temperatures (−4, 5, and 29 °C).

As the temperatures decrease, the thermal conductivity also declines. For example, all the samples exhibit at least 15% greater insulation at −4 °C. The bio-composites offer excellent thermal insulation properties at both low and high temperatures.

### 3.4. Sound Transmission Loss (STL)

**Figure 6:**
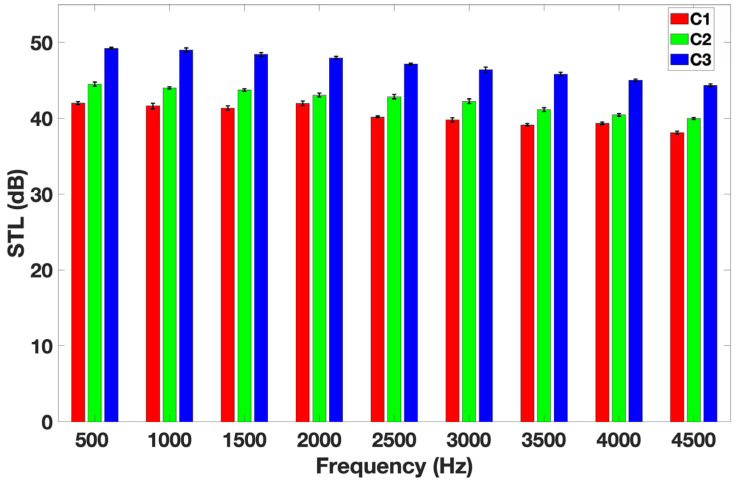
STL values versus frequency.

Figure 6 shows the effect of Ma on STL.

As seen in Figure 6, the STL value increases with Ma. STL values for sample C1 lie between 38.1 and 42.2 dB, whereas for sample C3, it ranges from 44.3 to 50.3 dB for Ma values of 748 g/m^2^ and 1228 g/m^2^, respectively. An increase in fibers per unit area results in a higher Ma, leading to lower apparent density and, thus, improved noise reduction. Many researchers have found the same result [27,44]. Several studies were conducted on the STL values of natural and synthetic fibers [27,44,45,46,47,48]. For example, Islam et al. measured STL values of banana, bamboo, and hemp composites with synthetic fibers [44]. They found that bamboo fibers combined with synthetic fibers offered the best STL values, ranging from 4 to 26 dB for an Ma of 964 g/m^2^, compared to banana and hemp fibers. For a banana composite with synthetic fibers, they found STL values ranging from 0 to 13 dB, which are less effective than those of milkweed bio-composites. Compared to milkweed bio-composites, kapok fibers also have a hollow structure. Karthik et al. obtained 40 and 45 dB STL values for an Ma of 700 and 900 g/m^2^ for composites made of cotton and milkweed fibers in a 40/60 weight ratio [27]. These values are comparable to the STL values measured in this study.

Hollow fibers offer a better sound reduction than other natural fibers (banana and hemp) and synthetic fibers. This could be due to their large lumen, which promotes greater entrapment of air [49,50]. Milkweed fibers exhibit a thin wall of 1.27 μm, while the lumen occupies a volume of 75% [51]. Consequently, it is easier for sound waves to interact with the fibers.

### 3.5. Tensile and Flexural Properties

Table 4 presents the weight of milkweed and PLA fibers for tensile and flexural samples.

In terms of absolute mass, there is more milkweed in sample STC3 compared to samples STC1 and STC2, even though the relative proportions remain the same. The same can be said for samples SFC1, SFC2, and SFC3.

**Figure 7:**
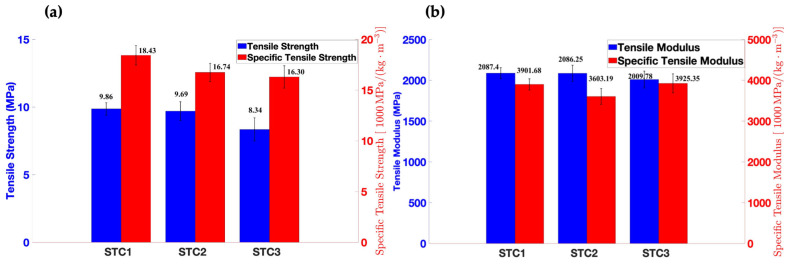
The tensile properties of STC1, STC2, and STC3 are represented in (**a**) bar graphs of tensile strength and specific tensile strength and (**b**) bar graphs of modulus and specific tensile modulus.

The tensile properties of milkweed/PLA bio-composites are exhibited in Figure 7.

As shown in Table 4 and Figure 7, tensile strength and modulus decrease with the increase in milkweed’s absolute mass increase. Samples STC1 and STC3 contain 3.50 g and 5.80 g of milkweed fibers, respectively. Adding 65% of milkweed fibers leads to a decrease of 15% in tensile strength and 3% in modulus. The same trend was reported by Reddy et al. for a milkweed/PP composite; they found that increasing the proportion of milkweed leads to a decrease in tensile strength [26]. Furthermore, the decrease in tensile strength and modulus may be related to the number of layers in the bio-composites. As shown in Figure 2, sample C1 demonstrates better dispersion between milkweed and PLA fiber than sample C3. The same trend was found by Mylsamy et al. They noticed that the tensile properties of a bio-composite are mainly affected by fiber orientation and fiber bonding [52]. Therefore, the more layers there are, the lower the tensile properties will be. For all the above explanations, the tensile strength and modulus are lower for sample C3 than for samples C1 and C2.

Notwithstanding this, the low density of the bio-composites leads to higher specific tensile properties. Indeed, the specific modulus of samples STC1, STC2, and STC3 are 3901.68 MPa, 3603.19 MPa, and 3925.35 MPa, respectively. Sample STC3 is 9% higher than sample STC2 and exhibits specific tensile properties similar to sample STC1 but with a lower density. Robert et al. found specific modulus values between 2114 MPa and 3106 MPa for a milkweed/PP composite of a 50/50 weight ratio [25]. Sample STC3 provides a specific modulus that is 26% and 86% higher compared to those reported by Robert et al.

Therefore, as previously discussed, it can be said that sample STC3 shows a better rigidity–density ratio compared to the other samples. According to Sapuan et al., the specifications required for automotive dashboards are a density of less than 1180 kg/m^3^, a tensile strength of at least 25 MPa, and a Young’s modulus of at least 2300 MPa [29].

Hence, sample STC3 conforms to the specifications and can be used as an automotive dashboard. Nevertheless, its tensile strength remains inferior but close to the requirement.

**Figure 8:**
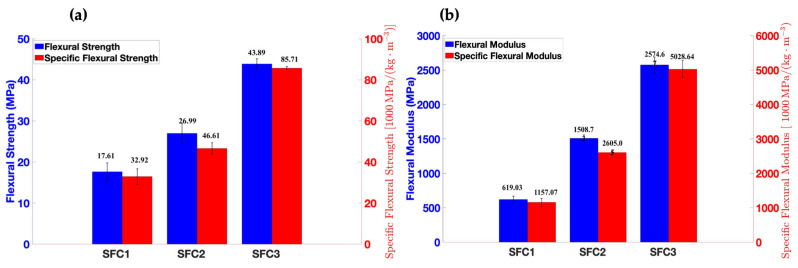
The flexural properties of SFC1, SFC2, and SFC3 are represented in (**a**) bar graphs of flexural strength and specific flexural strength and (**b**) bar graphs of modulus and specific flexural modulus.

The flexural properties of milkweed/PLA bio-composites are exhibited in Figure 8.

As seen in Figure 8, flexural strength and modulus increase with the increase in the milkweed’s absolute mass. The 5% increase in milkweed’s absolute mass in sample SFC2 results in a 53% flexural strength and a 144% improvement in flexural modulus compared to sample SFC1. The same trend was observed by Shibata et al. and Manalo et al. for composites made from biodegradable resin/bamboo and bamboo fiber/polyester composites [53,54]. Both studies found that the highest flexural modulus was obtained for composites with more bamboo fibers. Contrary to tensile properties, flexural properties increase with the number of layers. This is due to the nature of the stress. Whereas tensile strength is strongly dependent on fiber orientation, flexural strength is not. Comparing our values with those of Robert et al., we found flexural modulus values between 1600 MPa and 2200 MPa for a milkweed/PP composite of a 50/50 weight ratio [25]. Sample SFC3 provides a flexural modulus that is 17% and 60% higher than what Robert et al. reported.

As for tensile properties, the low milkweed density enhances the flexural strength and modulus. In fact, SFC1, SFC2, and SFC3 have specific flexural strength values of 17.51 MPa, 26.99 MPa, and 43.89 MPa, respectively. In addition, they show specific flexural modulus values of 1157.07 MPa, 2605.05 MPa, and 5028.64 MPa, respectively. SFC3 has a 160% increase in specific flexural strength and a 335% increase in specific flexural modulus compared to SFC1.

One reason that milkweed bio-composites present better mechanical properties than those found by Robert et al. is the nature of the process. Robert et al. made the nonwovens using a carding machine, while the PLA milkweed nonwovens were produced with an air-laying machine. Gharehaghaji et al. showed that their milkweed fibers were damaged as they passed through the carding cylinders [30].

Thus, even though sample C3 is the least homogeneous, it has the best mechanical-property-to-density ratio and better mechanical properties than those of a milkweed/PP composite with a 50/50 weight ratio.

### 3.6. Dynamic Mechanical Analysis

Table 5 presents the storage modulus of the bio-composites at varying temperatures from −30 °C to 60 °C.

The storage modulus E′ shows no variation for the three bio-composites. It remains constant over a temperature range from −30 °C to 60 °C.

### 3.7. Contact Angle

Table 6 shows the contact angle for the bio-composites.

Like PLA, milkweed is a hydrophobic fiber due to its chemical composition: 40–45% cellulose, 35–40% hemicellulose, 15% lignin, 3% free sugars, and 3% wax [20].

Blending these two fibers results in a hydrophobic material having a contact angle close to 180°. Lim et al. studied the wettability of kapok fibers with water. They measured a contact angle of 117° [55]. By the same token, Atmakuri et al. obtained a contact angle of 58° and 70° for hemp and flax fibers, respectively [56]. Therefore, milkweed bio-composites demonstrate higher hydrophobic properties than kapok and flax fibers.

## 4. Conclusions

In this paper, bio-composites made of milkweed and PLA fibers were processed by thermocompression to be employed as dashboards. From the study of their microstructure, thermal conductivity, sound transmission loss, and mechanical properties, the following conclusions have been drawn:

The hollow structure of milkweed fibers is not significantly affected by high-pressure molding. In fact, the bio-composites showed thermal conductivity values inferior to 43.36 mW/m·K, which remain much lower than those of other natural fibers such as *Calotropis procera* and bagasse. In addition, milkweed bio-composites exhibited STL values inferior to those of composites made from kapok, banana, and hemp fibers. These properties of thermal conductivity and STL are due to the hollow structure of milkweed.

As for the mechanical properties, it has been shown that the tensile strength and modulus are strongly dependent on fiber orientation in the bio-composites; they decrease with an increase in the number of nonwoven layers. Even if sample C3 has the least homogeneous structure, it exhibits the best mechanical-property-to-density ratio with values that conform to the specifications required for automotive dashboards.

All the bio-composites present good hydrophobic surfaces with contact angle values near 179°, superior to those made of kapok fibers. The smooth wall of milkweed fiber provides softness and a brightness aspect to the bio-composites.

Based on all the previous considerations, bio-composite C3 has all the required properties for use as a dashboard for car interiors. Nevertheless, its tensile strength remains inferior but close to the requirement; hence, improving the interface between PLA and milkweed fibers should counteract this.

To confirm that bio-composite C3 can be used as a dashboard, further investigations must be conducted, such as studying its wear and tear resistance, thermal stress, and resistance to ultraviolet light.

## Figures and Tables

**Table 1:** Properties of the fibers.

Fiber	Fineness (dtex)	Fiber Length (mm)	Fiber Diameter (μm)	Density (g/cm^3^)
MW	0.84–2.2	25 ± 3	22 ± 6	0.30 [16]
PLA	1.5 *	51 ± 0.4	12.2 ± 1	1.28

* Datasheets from companies.

**Table 2:** Composition of the bio-composites.

Sample	Number of Layers	Milkweed Fiber Mass (g)	PLA Fiber Mass (g)
C1	6	52.17	17.39
C2	7	56.84	18.95
C3	10	85.60	28.53

**Table 3:** The physical properties of the bio-composites: mass per unit area (Ma), thickness (TH), and density.

Sample	Mean Ma (g/m^2^)	SD * Ma (g/m^2^)	Mean TH (mm)	SD * TH (mm)	Density (kg/m^3^)
Bio-composite 1 (C1)	748.76	0.35	1.40	0.33	534.83
Bio-composite 2 (C2)	815.73	0.39	1.41	0.25	578.53
Bio-composite 3 (C3)	1228.49	1.58	2.40	0.42	511.87

* Standard deviation.

**Table 4:** Weight of milkweed and PLA fibers for tensile and flexural samples.

Tensile and Flexural Samples	Weight of Milkweed (g)	Weight of PLA (g)
Sample Tensile C1 (STC1)	3.50	1.17
Sample Tensile C2 (STC2)	3.86	1.29
Sample Tensile C3 (STC3)	5.80	1.99
Sample Flexural C1 (SFC1)	0.37	1.12
Sample Flexural C2 (SFC2)	0.39	1.13
Sample Flexural C3 (SFC3)	0.60	0.20

**Table 5:** Storage modulus of the bio-composites.

Samples	Storage Modulus (E′) (MPa)
C1	1273 ± 80
C2	1190 ± 87
C3	1100 ± 77

**Table 6:** Contact angle values of the bio-composites.

Samples	Contact Angle (°)
C1	179.7 ± 0.3
C2	179.8 ± 0.1
C3	179.7 ± 0.1

## Data Availability

The original contributions presented in the study are included in the article, further inquiries can be directed to the corresponding author.
